# Association of *ADRB2* Gene Polymorphisms with Sporting Ability and Human Body Functions: A Literature Review

**DOI:** 10.3390/genes17101311

**Published:** 2026-10-21

**Authors:** Xun Li, Sergey P. Levushkin, Marek Kruszewski, Maksim O. Aksenov

**Affiliations:** 1 School of Foreign Languages, Anyang Institute of Technology, West Section of Huanghe Avenue, Anyang 455099, China; li.xun@ayit.edu.cn; 2 Research Institute of Sports and Sports Medicine, Russian University of Sport “GTSOLIFK”, Sirenevy Boulevard 4, Moscow 105122, Russia; levushkin.sp@gtsolifk.ru; 3 Department of Physical Education, Faculty of Individual Sports, Jozef Pilsudski University of Physical Education in Warsaw, Marymoncka Street 34, 00-968 Warsaw, Poland; dr.makrus@wp.pl; 4 Department of Physical Education, Plekhanov Russian University of Economics, Stremyanny Lane 36, Moscow 115093, Russia; 5 Laboratory of Sports Genetics, Dorzhi Banzarov Buryat State University, Smolina Street 24a, Ulan-Ude 670000, Russia; 6 Department of Theoretical Foundations of Physical Culture, Sports and Health, Cherepovets State University, Lunacharsky Avenue 5, Cherepovets 162600, Russia

**Keywords:** sport, training, trainability, gene, polymorphism, *ADRB2*, obesity, long-lived individuals, rs1042713, rs1042714

## Abstract

*ADRB2* encodes the β2-adrenergic receptor, which is involved in regulating cardiovascular and respiratory function and lipolysis. This narrative review (PubMed/MEDLINE, Scopus and Web of Science Core Collection, to 8 October 2026; Preferred Reporting Items for Systematic Reviews and Meta-Analyses (PRISMA) 2020-adapted flow diagram) summarises 48 primary association studies of *ADRB2* polymorphisms, mainly *Arg16Gly* (rs1042713) and *Gln27Glu* (rs1042714), with athletic status, obesity, longevity, cardiovascular disease, asthma and chronic obstructive pulmonary disease (COPD); assesses their methodological quality; and critically examines meta-analyses. In athletes, mostly nominal associations link *Gly16* and *Glu27* with speed–strength status and *Arg16Gly* with endurance status, but a meta-analysis did not confirm an *ADRB2* association with runner and cyclist status. Obesity and diet-response associations are inconsistent, varying by population and sex. The rs1042718–rs1042719 haplotype was associated with longevity only in Han Chinese men, and *Glu27Glu* with increased myocardial infarction risk only in a subgroup with a specific angiotensin I-converting enzyme (*ACE*) genotype. In meta-analyses, *Glu27* is not associated with COPD risk in Asian or Caucasian populations. About half of the studies had fewer than 500 participants, almost none have been replicated, and many lack correction for multiple comparisons. The *ADRB2* gene cannot serve as a tool for predicting athletic performance or for selection; its contribution should be studied as part of multimarker panels.

## 1. Introduction

The beta-2 adrenergic receptor gene (*ADRB2*) contains no introns. The gene encodes the beta-2 adrenergic receptor, which mediates the effects of adrenaline, and its polymorphisms are associated with individual sensitivity to adrenaline. The gene has also been linked to muscle hypertrophy and anabolic processes in the human body. Acting through beta-2 adrenoceptors, adrenaline contributes to the regulation of cardiac activity and blood flow, which supply oxygen and nutrients to working muscles; *ADRB2* polymorphisms are considered possible modifiers of these responses, although the available evidence is associative. Activation of beta-2 adrenoceptors in bronchial smooth muscle causes bronchodilation; it has been suggested that *ADRB2* variants may modulate this response and thereby pulmonary function during prolonged aerobic exercise, but this hypothesis requires testing. In adipose tissue, beta-2 adrenergic receptors stimulate the breakdown of triglycerides into free fatty acids and glycerol, thereby mobilising fat as a fuel during training [1]. Polymorphisms of this gene have also been proposed as possible modifiers of weight loss on diets with different carbohydrate contents [2].

Associations have been reported between *ADRB2* polymorphisms and indices of several body systems, as well as endurance in athletes. It has been suggested that these associations reflect the role of the beta-2 adrenoceptor in regulating stroke volume and cardiac output, which determine the ability to sustain a given power output for long periods; however, a causal relationship has not been established. Pharmacological agonists of the beta-2 adrenergic receptor (β2-agonists) are used to enhance performance owing to their anabolic, lipolytic and ergogenic effects, as this receptor plays a central role in energy expenditure [3]. β2-Agonists are included in the World Anti-Doping Agency (WADA) Prohibited List as a separate class (S3, beta-2 agonists), and some of them (e.g., clenbuterol) are also classified as anabolic agents (S1). The *ADRB2* polymorphisms *Arg16Gly* (rs1042713) and *Gln27Glu* (rs1042714) have attracted the most interest in studies of human phenotypes and athletic performance.

## 2. Materials and Methods

### 2.1. Study Design

This article is a narrative literature review summarising published evidence on the associations of *ADRB2* gene polymorphisms with athletic performance and with metabolic, cardiovascular and respiratory phenotypes in humans. The review covers four thematic areas, which correspond to Section 3, Section 4, Section 5 and Section 6 of this article: (1) athletic status and performance in endurance and speed–strength sports, including cardiorespiratory adaptations to exercise; (2) obesity, body composition and the response to weight-loss diets; (3) ageing, longevity and cardiovascular disease; and (4) asthma and chronic obstructive pulmonary disease (COPD), including the response to β2-agonist therapy. The evidence was synthesised narratively; no quantitative synthesis (meta-analysis) was performed. This review is based exclusively on previously published data; therefore, ethical approval and informed consent were not required. The bibliography and in-text references were prepared using Zotero (Corporation for Digital Scholarship, Vienna, VA, USA) and Overleaf (Writelatex Ltd., London, UK).

### 2.2. Literature Search

The following electronic databases were searched: PubMed/MEDLINE, Scopus and Web of Science Core Collection. The search terms reflected the scope of the review and combined the gene name and the polymorphisms of interest with the phenotypes addressed, using the Boolean operators AND and OR: (“*ADRB2*” OR “beta-2 adrenergic receptor” OR “β2-adrenoceptor” OR “rs1042713” OR “*Arg16Gly*” OR “*Gly16Arg*” OR “rs1042714” OR “*Gln27Glu*” OR “*Glu27Gln*” OR “rs1042718” OR “rs1042719”) AND (“sport” OR “athlete” OR “athletic performance” OR “exercise” OR “training” OR “trainability” OR “endurance” OR “obesity” OR “body mass index” OR “body composition” OR “diet” OR “weight loss” OR “insulin resistance” OR “longevity” OR “long-lived individuals” OR “ageing” OR “aging” OR “life expectancy” OR “cardiovascular disease” OR “hypertension” OR “myocardial infarction” OR “coronary heart disease” OR “depression” OR “anxiety” OR “asthma” OR “COPD” OR “chronic obstructive pulmonary disease”).

PubMed/MEDLINE was searched across all fields (All Fields) without restrictions on date, language or publication type, i.e., from the inception of the database to the date of the last search. The last search was performed on 8 October 2026; the specified search string retrieved 1814 records in PubMed. Scopus, Web of Science Core Collection and the reference lists of the selected articles were used for a supplementary search for publications not retrieved by the main search string or not indexed in MEDLINE. This supplementary search identified 5 primary studies included in the review (one of them was published in a journal not indexed in MEDLINE); the number of records screened in the supplementary search was not recorded. Duplicates were identified by PubMed identifier (PMID) and digital object identifier (DOI), as well as by matching title, year of publication and abstract, followed by manual verification; 6 duplicates were removed (repeated records of the same publications in PubMed, including a preprint and its published version).

### 2.3. Eligibility Criteria and Characteristics of the Included Literature

The following inclusion criteria were applied: (1) studies conducted in humans; (2) articles reporting original data; (3) genotyping of *ADRB2* variants and analysis of their associations with at least one phenotype from the four thematic areas of the review (Section 2.1); and (4) articles published in peer-reviewed journals indexed in the databases searched, in English. Exclusion criteria were as follows: reviews, meta-analyses, editorials, commentaries, letters, case reports and preprints; animal, in vitro and in silico studies; studies in which *ADRB2* variants were not genotyped (pharmacological and physiological studies, gene expression and methylation studies, and studies of other genes only); studies of phenotypes outside the four areas of the review (e.g., oncological diseases); and descriptive (allele frequencies only) and methodological studies. Reviews, meta-analyses and other publications without original data were used as supplementary sources to provide context. Systematic reviews and meta-analyses on the genetics of athletes whose PubMed records do not mention *ADRB2* or its variants were not retrieved by the main search string; they are discussed in Section 3. The review addresses the associations of *ADRB2* polymorphisms—primarily *Arg16Gly* (rs1042713) and *Gln27Glu* (rs1042714), and also rs1042718 and rs1042719—with athletic status and performance, trainability, cardiorespiratory function, body composition, obesity and the response to diet, ageing and longevity, cardiovascular disease, asthma and COPD. Studies that analysed other genes, such as the fat mass and obesity-associated gene (*FTO*), the β3-adrenergic receptor gene (*ADRB3*) and the leptin receptor gene (*LEPR*), alongside *ADRB2* were included in the synthesis; studies focusing only on other genes were used as contextual sources, including for describing gene–diet interactions; in such cases, the gene studied is specified in the text.

**Figure 1:**
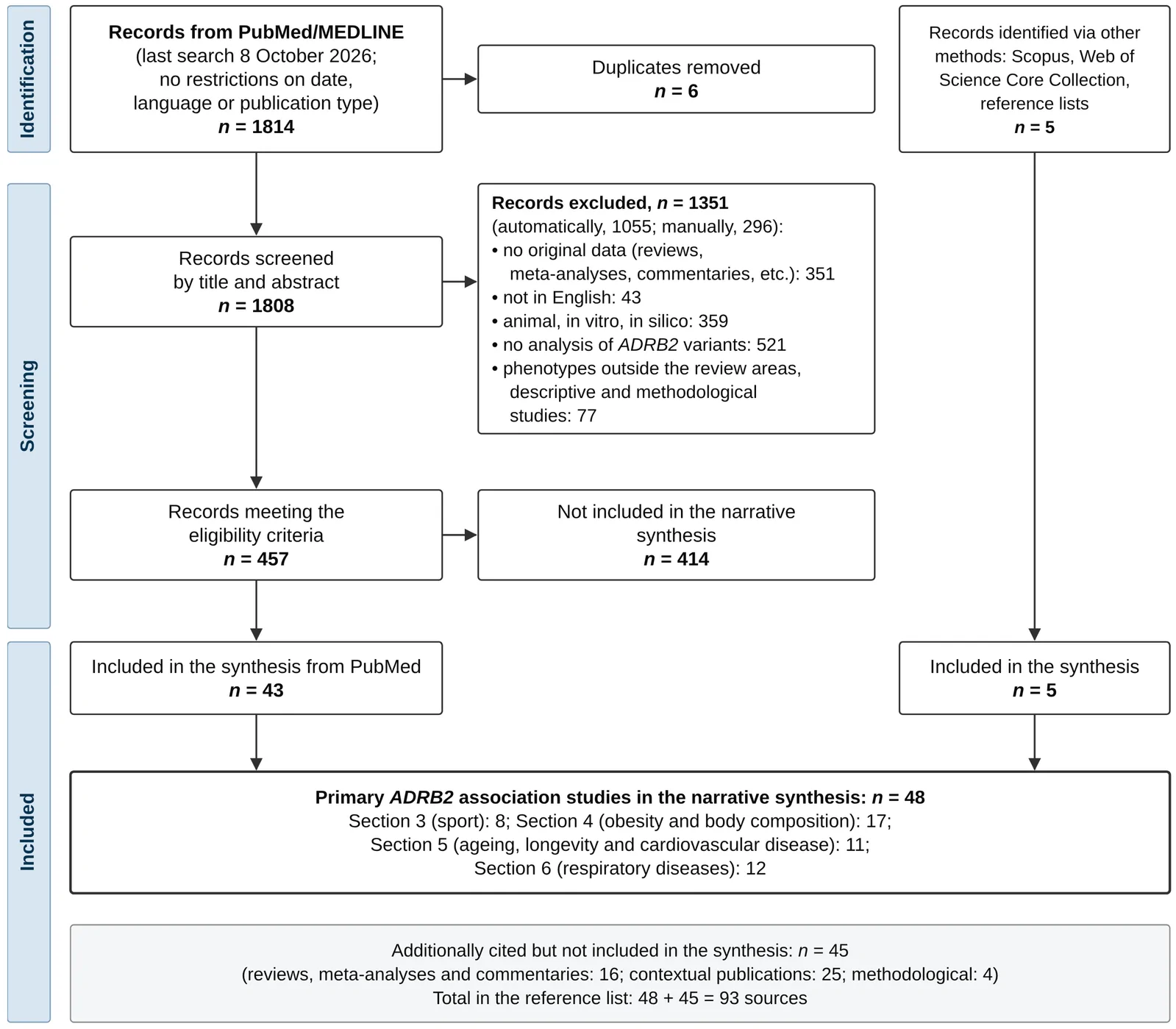
Flow diagram of the literature search and selection (adapted from the Preferred Reporting Items for Systematic Reviews and Meta-Analyses (PRISMA) 2020 statement [89]).

Selection was performed in two stages (Figure 1). In the first stage, the 1808 records remaining after removal of duplicates were screened by title and abstract. Publications without original data, publications in other languages and animal-only studies were identified using PubMed fields (publication type, language, and the Medical Subject Headings (MeSH) descriptors “Humans” and “Animals”); records whose title and abstract contained no indication of genetic analysis (polymorphism, genotype, allele, haplotype, variant, single-nucleotide polymorphism (SNP), rs number, etc.) were excluded as not containing an analysis of *ADRB2* variants (1055 records were excluded automatically in total). The remaining 753 records were screened manually by title and abstract; in case of uncertainty, a record was retained for the next stage. Initial screening was performed using Zotero according to predefined criteria. In total, 1351 records were excluded: no original data, 351; not in English, 43; animal, in vitro and in silico studies, 359; no analysis of genetic variants of *ADRB2*, 521; phenotypes outside the four areas of the review, as well as descriptive and methodological studies, 77. A total of 457 records met the eligibility criteria.

In the second stage, the authors selected studies for the narrative synthesis. Because this review is narrative rather than systematic, the synthesis is not exhaustive: it includes 43 primary studies from the PubMed search results and 5 studies identified by other methods (48 primary studies in total), selected as illustrating the main lines of *ADRB2* research in each thematic section; the remaining 414 eligible records were not examined individually. Such selection may be a source of bias, which should be taken into account when interpreting the conclusions. Decisions on the inclusion of studies in the synthesis were made by the authors; the screened records (PMIDs) and the decision for each record are listed in Table S1. The review protocol was not registered.

The review is based on 93 sources: 48 primary studies of *ADRB2* associations included in the synthesis; 41 sources used to provide context, namely reviews, meta-analyses and commentaries (*n* = 16) and other contextual publications (*n* = 25), such as general reviews and guidelines, studies and meta-analyses of other genes, and physiological, anthropometric, epidemiological and modelling studies without *ADRB2* genotyping; and 4 methodological and consensus sources, including the Single Nucleotide Polymorphism Database (dbSNP). Three sources are first cited in the Introduction [1,2,3]: a primary study [3] and a review and a commentary [1,2]. In Section 3, 20 sources are first cited [4,5,6,7,8,9,10,11,12,13,14,15,16,17,18,19,20,21,22,23]: primary studies [7,8,9,13,14,16,19,20], reviews [15,22,23], a meta-analysis [21] and contextual publications [4,5,6,10,11,12,17,18]. In Section 4, 23 sources are first cited [24,25,26,27,28,29,30,31,32,33,34,35,36,37,38,39,40,41,42,43,44,45,46]: primary studies [24,25,26,27,28,29,30,31,32,33,35,36,41,42,44,45] (in addition, study [3] is discussed in this section), a review [39], a meta-analysis [46] and contextual publications [34,37,38,40,43]. In Section 5, 23 sources are first cited [47,48,49,50,51,52,53,54,55,56,57,58,59,60,61,62,63,64,65,66,67,68,69]: primary studies [47,53,58,59,60,63,64,66,67,68,69], a review [61], meta-analyses [62,65] and contextual publications [48,49,50,51,52,54,55,56,57]. In Section 6, 19 sources are first cited [70,71,72,73,74,75,76,77,78,79,80,81,82,83,84,85,86,87,88]: primary studies [72,73,75,76,78,79,80,81,82,83,84,85], meta-analyses [74,86,87,88] and contextual publications [70,71,77]. Thus, the synthesis includes 8 primary studies from Section 3 (6 of them conducted in athletes [7,8,9,13,19,20]), 17 from Section 4, 11 from Section 5 and 12 from Section 6. The search and selection process is shown in Figure 1 (a simplified flow diagram adapted from the Preferred Reporting Items for Systematic Reviews and Meta-Analyses (PRISMA) 2020 statement [89]). Sources [89,90,91,92,93] (a systematic review with meta-analysis [90], discussed in Section 3; methodological and consensus publications; and the dbSNP database [89,91,92,93]) were used to describe the methods and interpret the results.

### 2.4. Data Extraction, Quality Assessment and Synthesis

For each study, the following information was extracted and is reported in the text where available: country or population, sample size and participant characteristics (athletes, non-athletes or patients), the polymorphisms, alleles, genotypes or haplotypes analysed, the phenotypes or outcomes assessed and the main quantitative results (allele, genotype and haplotype frequencies, *p* values, and odds ratios (ORs) with 95% confidence intervals (CIs)). Variant designations have been standardised. The traditional names *Arg16Gly* (rs1042713) and *Gln27Glu* (rs1042714) are used; in the literature, they also appear as *Gly16Arg* and *Glu27Gln*, and in the Human Genome Variation Society (HGVS) nomenclature relative to the reference sequence NM_000024.6 they correspond to c.46G>A (p.*Gly16Arg*) and c.79G>C (p.*Glu27Gln*) [93]. The A allele of rs1042713 encodes *Arg16* and the G allele encodes *Gly16*; the C allele of rs1042714 encodes *Gln27* and the G allele encodes *Glu27*. Genotypes are designated by amino acids, with the nucleotide genotype given at first mention (e.g., *Gly16Gly*, GG rs1042713). Only allele combinations reconstructed by haplotype analysis in the original studies are termed haplotypes (hyphenated, e.g., *Arg16-Gln27*); combinations of genotypes at two polymorphisms in the same individual (separated by a slash, e.g., *Arg16Gly*/*Gln27Gln*) are termed genotype combinations, even if they are referred to as haplotypes in the original study. The findings were grouped by thematic area and synthesised narratively; agreement and contradictions between studies were noted, and potential sources of heterogeneity (differences in the populations studied, study design, ethnicity, measurement scales, nutrition and sample size) were considered. Results with *p* values ≥ 0.05 or with 95% confidence intervals including 1 were interpreted as not statistically significant. Because candidate-gene studies usually test several alleles, genotypes (inheritance models), haplotypes and subgroups, a threshold of *p* < 0.05 without correction for multiple comparisons increases the risk of false-positive results. Therefore, results with *p* < 0.05 obtained without such a correction (e.g., the Bonferroni correction, in which the threshold is 0.05/*k*, where *k* is the number of tests) or in subgroup analyses are described as nominal and requiring replication; for comparison, genome-wide association studies use a threshold of *p* < 5 × 10^−8^, which corresponds to a correction for approximately 10^6^ independent tests [92]. The genetic association studies included in the review are observational: the genotype is not assigned by the investigator, including in studies with dietary interventions. Therefore, the associations identified were interpreted as statistical associations rather than as evidence of a causal effect of *ADRB2* variants on the phenotype. The genotype is not altered by the phenotype, which rules out reverse causation; however, an association may reflect linkage disequilibrium with another, causal variant, population stratification, confounding or a false-positive result.

The methodological quality of the primary studies was assessed against five criteria, by analogy with systematic reviews in sports genetics [90]: sample size (at least 500 participants), homogeneity of the sample with respect to ethnic origin, conformity of the genotype distribution to Hardy–Weinberg equilibrium (HWE), objective phenotype definition (for athletes, performance level or physiological criteria) and replication of the finding in an independent sample. Full texts of the articles were used or, when these were unavailable, PubMed abstracts (8–9 October 2026). The results are presented in Table 1.

As Table 1 shows, the evidence base for athletes is limited. Of the six studies of athletes, only two had a sample of more than 500 individuals [7,9], and none was replicated in an independent sample. The study by Wolfarth et al. [9], in which athletes and controls were selected by maximal oxygen uptake (VO_2_max ≥ 75 and ≤50 mL·kg^−1^·min^−1^, respectively), can be considered the most rigorous in design. Overall, among the studies reviewed, findings were replicated in only two [33,58], and conformity to HWE was confirmed in fewer than one-third of the studies. Therefore, the associations of *ADRB2* with athletic traits should be regarded as preliminary.

## 3. Association of *ADRB2* with Sport

High levels of skeletal muscle strength and endurance in athletes result from various metabolic and physiological responses to exercise. Depending on the type and intensity of exercise, energy metabolism may shift towards anaerobic or aerobic pathways. The energy supply to working muscles is also influenced by nutrition, training methods and genetic factors.

Associations between gene polymorphisms and elite athletic performance are currently studied extensively by both geneticists and sports scientists [4,5,6]. For example, one study compared the frequencies of the *ADRB2 Arg16Gly* and *Gln27Glu* polymorphisms in athletes from sports requiring endurance, speed–strength qualities or high muscle power [7]. The aim was to test whether *Arg16Gly* and *Gln27Glu* genotypes are related to maximal strength and endurance. The study included 223 high-level Polish athletes (123 endurance-oriented athletes and 100 speed–strength athletes); the control group comprised 354 unrelated sedentary volunteers. The *Gly16* and *Glu27* alleles were significantly more frequent in speed–strength athletes than in controls (69.0% versus 59.7%, df = 1, *p* = 0.017 and 51% versus 41.5%, df = 1, *p* = 0.017, respectively). These differences are nominal: with two polymorphisms and two groups of athletes, the Bonferroni-corrected threshold would be 0.0125 or lower. The higher frequency of these alleles in speed–strength athletes indicates an association with athlete status in sports requiring high skeletal muscle strength. Because this was a case–control study, these data do not allow conclusions about the effect of the *Gly16* and *Glu27* alleles on achieving high performance in speed–strength sports. No association of the *Gly16* or *Glu27* alleles with endurance was found in athletes from aerobic sports.

In another study [8] involving endurance-oriented athletes, the minor *A* allele of rs1042713 (*Arg16*) was associated with faster race times in male habitual runners; however, this association was found only in a subgroup of 316 men and was nominal (*p* = 0.01; with 11 polymorphisms and three phenotypes, the Bonferroni-corrected threshold would be approximately 0.0015); no associations were found in the total sample of 438 participants.

Another study [9] included 313 elite athletes from aerobic cyclic sports and 297 sedentary men (control group) from North America, Finland and Germany. Genotype distributions differed significantly between the two groups (*p* = 0.03), suggesting an association between the *Arg16Gly* polymorphism and endurance. Because carriers of the *Gly16* allele were more frequent in the control group (*p* = 0.009), an association of the *Arg16* allele with a predisposition to endurance was suggested.

Success in sport depends on both genetic and environmental factors, including specialised training and nutrition. At present, genetic markers are regarded as potential tools for talent identification and for the prevention of diseases and injuries in athletes; however, their practical application requires further validation [10]. According to the consensus of experts in sports genetics, given the current state of knowledge, genetic tests should not be used to identify athletic talent or to select children and young athletes [91]. Therefore, individual gene variants, including *ADRB2*, cannot be used to identify athletes predisposed to rhythmic gymnastics, although they may be studied as part of multimarker panels.

Success in rhythmic gymnastics is largely determined by anthropometric characteristics, including low body mass and low body fat [11,12]. To determine whether genetic predisposition contributes to the gymnast phenotype, the frequencies of *ADRB2* polymorphisms associated with body mass were analysed in female athletes. In one study [13], 42 gymnasts competing at national and international level were compared with 42 controls. The *Gly16Gly* genotype (GG rs1042713) was found in 42.8% of the gymnasts and in 23.8% of the controls, whereas the *Arg16Arg* (AA) genotype was significantly less frequent in the gymnasts (7.1% versus 21.4%). The *Arg16Arg* genotype was more common in gymnasts with a higher body mass index (BMI), whereas the *Gly16Gly* genotype was associated with a lower BMI. Similarly, in a study of adolescents in Taiwan, girls with the *Gly16Gly* genotype had a lower BMI than those with the *Arg16Arg* genotype [14].

Long-term training induces functional and structural adaptations in athletes, particularly in the oxygen transport and cardiovascular systems. Muscle blood flow improves, enhancing the supply of oxygen and nutrients to tissues. Changes in pulmonary ventilation in response to aerobic exercise are also important because they facilitate the removal of carbon dioxide and other metabolites from working muscles. The association of the *Arg16Gly* polymorphism of the *ADRB2* gene with the cardiovascular and respiratory responses to exercise has been summarised in a review [15]. In healthy non-athletes, *Gly16Gly* homozygotes, compared with *Arg16Arg* homozygotes, had a greater stroke volume and cardiac output at rest, during short-term exercise (cycle ergometer exercise, isometric handgrip dynamometer exercise) and during recovery, and a lower total peripheral vascular resistance during isometric exercise. Differences in airway function in healthy individuals were not found at rest or during short-term exercise and were detected only after intense exercise (more sustained bronchodilation in the *Gly16* group and mild bronchoconstriction in the *Arg16* group) [15]. In a population sample of normotensive adults (non-athletes), *Gly16Gly* homozygotes had higher indices of left ventricular systolic function at rest, in particular ejection fraction (65.0% versus 62.5% and 62.6% in heterozygotes and *Arg16Arg* homozygotes, respectively, *p* = 0.01), whereas no such association was found in individuals with arterial hypertension [16]. These data were obtained in observational studies of non-athletes at rest and during short-term exercise. According to the authors of the review [15], an effect of the *ADRB2* genotype on the cardiovascular response to prolonged exercise has not been established, and its effect appears to be insufficient to alter the respiratory response to exercise in healthy individuals. Therefore, these data do not allow conclusions about the effect of the *Arg16Gly* polymorphism on endurance in athletes; extrapolation of these results to athletes requires confirmation in studies involving athletes performing prolonged exercise.

Several studies have examined in detail the genes and skeletal muscle structures involved in the metabolic basis of endurance in athletes. However, the mechanisms through which genetic variants are associated with endurance remain insufficiently clear: for example, the association of the rs12722 polymorphism of the collagen type V alpha 1 chain gene (*COL5A1*) with endurance running performance was not explained by differences in running economy [17]. Nevertheless, there is evidence that genetic markers may partly explain individual differences in trainability in response to prolonged aerobic exercise [18]. The *ADRB2 Arg16Gly* and *Gln27Glu* polymorphisms may be considered candidate markers of a predisposition to endurance. One study included 82 judoka from Poland and 26 from Croatia [19]. The heterozygous *Arg16Gly* genotype was more common among the most successful competitors; however, this difference was not statistically significant (*p* = 0.06). Therefore, these data do not demonstrate an effect of the *ADRB2 Arg16Gly* polymorphism on competitive performance in sports that require sustained power output, and the role of *ADRB2* as a marker of elite endurance performance requires confirmation.

The results of individual case–control studies should be considered alongside those of systematic reviews and meta-analyses. Studies of *ADRB2* in athletes and meta-analyses are summarised in Table 2. In a systematic review with meta-analysis of 43 studies (3938 athletes and 10,752 controls) [90], polymorphisms of the angiotensin I-converting enzyme (*ACE*), α-actinin-3 (*ACTN3*), peroxisome proliferator-activated receptor gamma coactivator 1-alpha (*PPARGC1A*), adenosine monophosphate deaminase 1 (*AMPD1*) and homeostatic iron regulator (*HFE*) genes, but not of *ADRB2*, were associated with the status of national- and international-level long-distance runners and road cyclists: the odds ratio was 0.99 (95% CI 0.67–1.46) for the *Gly16Gly* genotype (GG rs1042713) and 1.29 (95% CI 0.86–1.96) for the *Gln27Gln* genotype (CC rs1042714). These estimates were obtained from only two Spanish samples (223 athletes and 222 controls); therefore, their confidence intervals are wide and do not rule out a moderate effect. One of these samples was examined in study [20] (123 elite long-distance runners and cyclists and 122 controls; all participants were men of Caucasian origin): the distribution of *Arg16Gly* genotypes did not differ (*p* = 0.943), whereas the *Glu27Glu* genotype (GG rs1042714) was less frequent in athletes than in controls (13.8% versus 32.0%, *p* = 0.003); this difference remains significant after Bonferroni correction for the eight polymorphisms studied (threshold 0.05/8 ≈ 0.006). At the same time, the total genotype score for eight polymorphisms of five genes related to cardiorespiratory function, including *ADRB2*, did not differ between athletes and controls and did not distinguish elite athletes from controls [20]. The absence of an association of *Arg16Gly* with athlete status in [20,90] is inconsistent with the data of Wolfarth et al. [9], obtained in athletes from various cyclic sports from North America, Finland and Germany.

In the meta-analysis [21], 107 articles on 37 genes were selected from 4228 records; however, only two articles were found for each of the rs1042713 and rs1042714 polymorphisms, and a pooled analysis was performed only for *ACE* and *ACTN3* and revealed no statistically significant association with athlete status. The review [22] summarises data on at least 155 genetic markers of athlete status, including 41 markers identified in genome-wide association studies (GWAS); the association has been confirmed in at least two studies for only 31 markers and has not been replicated for 29 markers, which points to the possibility of false-positive results. *ADRB2* is classified among the genes whose variants are associated with human endurance [22,23]. In a systematic review of experimental studies in mice [23], endurance increased when the function of 31 genes was altered, including knockout of the *Adrb2* gene (the mouse orthologue of *ADRB2*) (running distance increased by 65%). This points to the involvement of the β2-adrenoceptor in the regulation of endurance but does not allow conclusions as to which *Arg16Gly* and *Gln27Glu* alleles are favourable for athletes. Thus, the meta-analysis [90] did not confirm an association of *ADRB2* polymorphisms with the status of endurance athletes, and in the meta-analysis [21] the data were insufficient to assess it; the positive results of individual studies [7,9,20] require replication in large independent samples of athletes of different ethnic origins.

The associations of *ADRB2* should be considered in a polygenic context. Calculations for 23 endurance-associated polymorphisms show that the probability of a single individual having all “favourable” genotypes is only 0.0005%, and that in 99% of people the overall profile differs from the typical one by no more than seven genotypes [6]. Therefore, the contribution of any single variant, including *Arg16Gly* or *Gln27Glu*, is small; however, even a total genotype score based on eight polymorphisms, including two *ADRB2* variants, did not distinguish elite athletes from controls [20]. The associations of *ADRB2* variants with phenotypes may depend on other genes and environmental factors: interactions of *ADRB2* with the *LEPR* gene for obesity risk in men [33,35], with the *ACE* compound genotype for myocardial infarction risk [68] and with regular exercise for health indicators in old age [53] have been described (Section 4 and Section 5). These interactions were identified in single samples and require replication.

## 4. Association of *ADRB2* with Obesity

The genetic factors associated with obesity remain a matter of debate. Some researchers have found an association between obesity and polymorphisms of the *ADRB2* gene [24,25], whereas others have been unable to detect such an association [26]. Reliable data on the relationship between gene polymorphisms and the response to different weight-loss diets are still insufficient, which limits the development of personalised diets that take genetic characteristics into account. A study in southern Brazil [27] included 109 obese adult premenopausal women who followed a seven-week weight-loss diet. The authors found no statistically significant differences between the weight-loss diets in carriers of different *ADRB2 Gln27Glu* (rs1042714) and *Arg16Gly* (rs1042713) genotypes. Nevertheless, carriers of the *Glu27* allele of the *ADRB2* gene had a statistically significantly lower mean body mass index than non-carriers (*p* = 0.006), and this difference was already present before the start of the diet. Because the genotype was not an intervention in this study, this result reflects an association in the studied sample and does not indicate a protective effect of the polymorphism. The *ADRB2* polymorphisms, like the variants of *ADRB3* and of the ghrelin and obestatin prepropeptide gene (*GHRL*) examined in the same study, did not modify weight loss on the diet [27].

In a Swedish population, the *ADRB2* rs1042713 polymorphism was strongly associated with central fat accumulation, systolic blood pressure, and serum leptin and triglyceride levels, but not with overall obesity [28].

In Saudi Arabia, a statistically significant association was found between the rs1042713 polymorphism of *ADRB2* and obesity [29]. However, studies in Asian (Japanese and Korean) populations found no association between *ADRB2* polymorphisms and obesity; these studies did not examine interactions between *ADRB2* polymorphisms and specific nutrients [30,31]. In contrast, a meta-analysis that included 10,404 participants genotyped for rs1042714 and 4328 genotyped for rs1042713 found no association of either polymorphism with obesity overall; however, the *Glu27* allele was significantly associated with obesity in Asians, Pacific Islanders and American Indians (groups with a low frequency of this allele), but not in Europeans [46].

In Malaysian adults, *ADRB2* rs1042713 was not significantly associated with obesity but was associated with insulin resistance. Carriers of the *Gly16* allele (G rs1042713) showed an increased risk of insulin resistance compared with subjects with the *Arg16Arg* (AA) genotype. Obese individuals carrying the *Gly16* allele had significantly higher levels of total cholesterol, low-density lipoprotein (LDL) and high-density lipoprotein (HDL) than subjects with the *Arg16Arg* genotype [32].

One study [33] examined the association of *ADRB2* (β2-adrenoceptor) polymorphisms with the risk of excess body fat in 4193 middle-aged and elderly Japanese individuals without diabetes (1911 women and 2282 men), with analyses stratified by sex. None of the polymorphisms studied showed a statistically significant individual effect, and haplotype analysis likewise revealed no associations of *ADRB2* polymorphisms with BMI [33]. However, analysis of gene–gene interactions using dimensionality reduction-based models demonstrated statistically significant interactions between the *ADRB2* polymorphisms and the *Gln223Arg* polymorphism of the *LEPR* gene for BMI and for the risk of obesity, defined as BMI ≥ 27 kg/m^2^; all of these interactions were found in men only [33]. The authors noted that the effect sizes associated with these interactions may be smaller in the population studied; heterogeneity of effect estimates between studies may be related to differences in study design, as has been shown for the insulin-induced gene 2 (*INSIG2*) [34]. The data presented confirm the previously reported interaction between the *LEPR Gln223Arg* and *ADRB2 Arg16Gly* polymorphisms in the risk of excess body mass and obesity, which in an independent sample was limited to men [35].

In one study of the *ADRB2* gene in obese Spanish women [36], the *Gln27Glu* polymorphism (rs1042714), but not *Arg16Gly* (rs1042713), was associated with differences in the response of body weight and body composition to a 12-week energy-restricted diet. Women carrying the *Glu27* (*G*) allele of rs1042714 showed a greater reduction in body weight than *Gln27Gln* (CC) homozygotes. Gene–diet interactions have also been described for variants of other genes: for example, in the 2-year Preventing Overweight Using Novel Dietary Strategies (POUNDS LOST) trial [37], participants with the *CC* genotype of rs2943641 (insulin receptor substrate 1 gene, *IRS1*) assigned to the high-carbohydrate, low-fat diet showed greater weight loss and a greater improvement in insulin resistance at 6 months than those with the *CT* and *TT* genotypes. In the same trial, carriers of the C allele of the rs1440581 variant near the protein phosphatase, Mg^2+^/Mn^2+^ dependent 1K gene (*PPM1K*) lost less weight on a high-fat diet than individuals without this allele [38]. These findings are consistent with the suggestion that people with different genetic backgrounds may respond differently to the same diet [39]. Understanding gene–nutrient interactions may help tailor diets to minimise the metabolic consequences of obesity, attenuate insulin resistance and reduce the risk of cardiometabolic diseases [40].

One study [41] assessed the association of *FTO* (rs9930501, rs9930506 and rs9932754) and *ADRB2* (rs1042713 and rs1042714) polymorphisms with the risk of obesity. The sample comprised 178 adult subjects (Malaysian Chinese, Malays and Indians) aged ≥18 years. No statistically significant difference was found between the polymorphisms under study and the diets applied (*p* > 0.05). Significant differences were observed between the ethnic groups that followed different diets. Malaysian Indian participants responded better to a low-calorie diet than Malaysian Chinese participants (*p* = 0.008). However, such differences between ethnic groups in the response to diet do not in themselves demonstrate a genetic predisposition to obesity and require further study.

Another study examined the associations of *ADRB2* polymorphisms with diet-induced weight loss, body mass, body composition and fat distribution in obese premenopausal women aged 25–45 years [42]. In this study, the *FTO* genotype was associated with body mass, which is consistent with previously reported data [43], and the *ADRB2* genotype was associated with body fat distribution [42]. Carriers of the *Gln27Gln* genotype (CC rs1042714) had a lower percentage of gynoid fat than carriers of the *Glu27Glu* genotype (*p* = 0.009); the difference relative to the *Gln27Glu* genotype (*p* = 0.050) is not statistically significant according to the criterion in Section 2.4. The *Gln27Gln* homozygotes also had a lower percentage of total fat and a higher percentage of lean body mass than *Glu27Glu* homozygotes (*p* = 0.018 and *p* = 0.019, respectively). In other studies, the *Glu27* allele was associated with obesity and increased BMI [3,44], and in a Swedish sample with obesity in women, whereas in men the association was inverse [45].

## 5. Association of *ADRB2* with Life Expectancy

In the Framingham Heart Study Offspring cohort, the *Gln27Glu* polymorphism (but not *Arg16Gly*) was associated with a number of age-related phenotypes and with longevity, and the direction of the association with longevity differed between age groups [47]. The authors of this study suggested that *ADRB2* gene polymorphisms may be systemic determinants of healthy ageing [47]. Data on the association of the *ADRB2* gene with longevity are still scarce. β-Adrenoceptor function has also been linked to certain aspects of psychological health. For example, altered β-adrenoceptor function or density, assessed mainly in lymphocytes, has been reported in panic disorder [48], hostility [49], psychomotor agitation [50], anxious tension [51] and depression [52].

A Chinese study of 877 people aged over 90 years combined phenotype, genotype and questionnaire data. Interactions between social and behavioural factors (regular exercise and social-leisure activities) and *ADRB2* genotypes were significantly and positively associated with health outcomes (cognitive function and self-reported health). Carriage of the minor alleles of rs1042718 or rs1042719 was also associated with fewer negative emotions in these participants [53].

Population ageing is a topical research subject worldwide for gerontologists, geneticists, physiologists, exercise therapy specialists and others. Genetics and environmental influences are the main factors in human ageing [54]. The functions of *ADRB2* (the beta-2 adrenergic receptor gene) require detailed analysis, and several researchers have sought associations between *ADRB2* and ageing-related phenotypes.

In recent decades, attempts have also been made to estimate the contribution of genetics to longevity. Genetic influences have been found to increase with age, particularly after the age of 60 years [55]. Genetic factors may contribute to longevity directly, by mediating signalling pathways involved in ageing, or indirectly, by buffering the harmful effects of other factors [56,57].

A study of the Han Chinese population [58] examined the association of *ADRB2* with lifespan in 963 long-lived individuals (mean age, 101 years) and 1028 young people (mean age, 44 years). DNA from long-lived participants older than 95 years was genotyped and analysed for association with longevity. The researchers identified two synonymous SNPs of *ADRB2* (rs1042718 and rs1042719) that were significantly associated with longevity in men. No such associations were found in women, which may indicate a smaller effect size in women. In men, the A-C haplotype (rs1042718 A and rs1042719 C) was more frequent in long-lived individuals than in young people (haplotype frequency 0.332 versus 0.250, OR = 1.49, *p* = 0.0007). The association was found in two geographically isolated Han Chinese samples and remained significant after Bonferroni correction, and the A-C haplotype reduced the translational efficiency of the β2-adrenoceptor in vitro [58]; however, it has been described only in men of a single ethnic group and requires confirmation in other populations.

Several polymorphisms have been identified in *ADRB2*, including *Arg16Gly* (rs1042713) and *Gln27Glu* (rs1042714), and a number of studies have examined their associations with cardiovascular diseases [59,60]. *ADRB2* polymorphisms have been linked to several cardiovascular diseases, including hypertension, heart failure and myocardial infarction [61,62]. *ADRB2* plays an important role in modulating coronary artery dilation in humans. In a prospective study of 25,224 women, no associations of *ADRB2* polymorphisms with myocardial infarction were found in the cohort as a whole; only in women of Caucasian origin was the *Gly16-Gln27-Thr164* haplotype associated with a reduced risk of myocardial infarction (hazard ratio [HR] = 0.75, 95% CI 0.58–0.97) [63]. In study [64] and in a meta-analysis of seven case–control studies (6843 participants) [65], *ADRB2* polymorphisms were associated with an increased risk of myocardial infarction and coronary heart disease, whereas other researchers have reported the opposite [66,67].

In the Framingham Heart Study Offspring cohort (*n* = 1500, follow-up of approximately 36 years), a multimarker analysis was performed of the associations of the rs1042713 and rs1042714 polymorphisms of the *ADRB2* gene and 11 polymorphisms of the *ACE* gene with the risk of myocardial infarction (MI) [68]. A 3.3-fold increase in MI risk (*p* = 0.021, above the threshold of 0.005 adopted by the authors with Bonferroni correction for the main analyses) in carriers of the *Glu27Glu* genotype (GG rs1042714) was found not as a general effect of this genotype but only in individuals with the GG/GG compound genotype for the *ACE* polymorphisms rs4363 and rs12449782 (compared with the *Gln27Glu* genotype with the same *ACE* genotype); no associations of the polymorphisms studied with all-cause mortality were found in this cohort [68]. In the Cardiovascular Health Study, the previously reported association of the *ADRB2* genotype with coronary heart disease in older adults was not explained by blood pressure level, subclinical atherosclerosis or antihypertensive therapy [69]. An indirect link between the *ADRB2* gene and life expectancy via cardiovascular disease was suggested by the authors of study [47]; however, this suggestion is based on conflicting association data and remains a hypothesis.

## 6. Association of *ADRB2* with Respiratory Function

Asthma is characterised by chronic airway inflammation, bronchial hyperresponsiveness and respiratory symptoms [70]. Long-acting β2-adrenoceptor agonists in combination with inhaled glucocorticoids are part of standard maintenance therapy for asthma [71]. Data on the associations of *ADRB2* polymorphisms with airway diseases are ambiguous. In two large population-based studies in Denmark (8971 and 53,777 individuals), the *Thr164Ile* variant (rs1800888) was associated with reduced lung function and an increased risk of COPD [72]; in a case–control study in North India (410 patients with asthma and 414 controls), the *Gly16* allele was associated with a reduced risk of asthma, whereas the *Gln27Glu* polymorphism was not associated with asthma [73]. In a meta-analysis of three studies (960 children with asthma), the *Arg16Arg* genotype was associated with a better bronchodilator response to inhaled β2-agonists (OR = 1.77, 95% CI 1.01–3.1), whereas the *Gln27Glu* polymorphism was not associated with the response [74].

A study in Kazakhstan [75] included 150 individuals: 70 patients with asthma and 80 healthy controls. Allele and genotype frequencies of the *Arg16Gly* and *Gln27Glu* polymorphisms did not differ significantly between patients and controls. Differences were reported for the frequencies of the *Arg16Gly*/*Gln27Gln* and *Arg16Gly*/*Gln27Glu* genotype combinations (*Arg16Gly* heterozygotes with the *Gln27Gln* and *Gln27Glu* genotypes, referred to as haplotypes in the original study) (OR = 2.12, 95% CI 0.87–5.16 and OR = 2.25, 95% CI 0.89–5.67, respectively); however, both 95% CIs include 1, and these differences therefore cannot be considered statistically significant. The frequencies of the *Arg16* allele and of the *Arg16Arg* genotype were higher in patients with uncontrolled asthma than in the controls (χ^2^ = 5.17, df = 1, *p* = 0.02 and χ^2^ = 5.36, df = 1, *p* = 0.02, respectively). These results indicate a possible association of the *Arg16Gly* polymorphism with uncontrolled asthma, whereas the higher frequency of these genotype combinations in patients with asthma requires confirmation.

A study in South India [76] examined the association of the two most common *ADRB2* polymorphisms, *Arg16Gly* and *Gln27Glu*, with asthma in 112 patients and 127 healthy volunteers. Neither polymorphism was associated with asthma individually; however, the combination of the *Gly16Gly* (GG rs1042713) and *Gln27Glu* (CG rs1042714) genotypes, designated by the authors as the GG-CG haplotype, was associated with a 3.1-fold increase in asthma risk (*p* = 0.02).

Associations of *ADRB2* with chronic obstructive pulmonary disease (COPD) have also been studied. COPD is usually progressive, and exacerbations of respiratory symptoms are often associated with environmental pollution, bacterial or viral respiratory infections and other factors [77]. Data on the association of *ADRB2* polymorphisms with the response to inhaled β2-agonists during COPD exacerbations are contradictory [78,79]. The Rotterdam Study [80] examined 1053 patients with COPD to determine whether two functional *ADRB2* SNPs, *Arg16Gly* (rs1042713) and *Gln27Glu* (rs1042714), are associated with the risk of exacerbations in patients receiving inhaled β2-agonists. Both polymorphisms (rs1042713 and rs1042714) were associated with a reduced risk of COPD exacerbations in users of inhaled β2-agonists. In addition, among β2-agonist users, carriers of the *Arg16-Gln27* haplotype had a significantly lower risk of exacerbation of COPD than carriers of the *Gly16-Glu27* haplotype.

Another study examined the association of *ADRB2 Arg16Gly* and *Gln27Glu* with the risk of severe COPD exacerbations and found an increased risk in carriers of *Arg16* and *Gln27* [81]. These results contrast with those of the Rotterdam Study [80], in which the *Arg16-Gln27* haplotype was associated with a reduced risk of exacerbations in current users of inhaled β2-agonists; the reasons for this discrepancy remain unclear.

In an Egyptian study of patients with COPD (*n* = 61), *ADRB2* genotypes were associated with COPD exacerbations. Genotypes and haplotypes containing *Arg16* were associated with frequent exacerbations of COPD [82].

Data on the association of the *Glu27* variant (rs1042714) with COPD differ by population and phenotype. In European cohorts, the *Gln27Glu* polymorphism was associated with the frequency of COPD exacerbations, but in opposite directions [80,81], whereas in the multicentre Prevention of Exacerbations with Tiotropium in COPD (POET-COPD) trial (5125 patients) it did not affect the risk of exacerbations [79]. In an Egyptian sample, carriage of the *Glu27* allele was associated with a reduced risk of COPD (*p* = 0.002) [82]. In Japanese studies, the *Arg16* allele and haplotypes containing *Gln27* were associated with the response to β2-agonists [83,85]; an association of the *Glu27* variant itself with COPD was not reported in these studies. In a meta-analysis of 16 studies (85,381 participants), the *Gln27Glu* polymorphism was not associated with COPD risk (OR = 1.00 for homozygotes and 0.94 for heterozygotes, the 95% CIs included 1), including in separate analyses of Asian, Caucasian and African populations [86]; the authors of an earlier meta-analysis reached the same conclusion, noting differences in genotype distribution between Caucasian and Asian populations [87]. An association specific to Asian populations has been reported in a meta-analysis for a different polymorphism: the *Arg16* allele was associated with COPD risk in smokers of Asian origin (OR = 1.27, 95% CI 1.03–1.57), but not in individuals of European origin [88]. Thus, the association of *Glu27* with COPD has not been confirmed by meta-analyses in either Asian or other populations, and individual positive findings (COPD exacerbations in European cohorts and COPD risk in an Egyptian sample) require replication.

In summary, several studies have assessed the association of *ADRB2* polymorphisms with the response to β2-agonist treatment, with highly contradictory results [83,84,85]. This variability may reflect differences in the populations studied, study design, ethnicity, measurement scales, nutrition and sample size.

## 7. Conclusions

*ADRB2* polymorphisms have been associated with sporting ability and with the function of several body systems, including the cardiovascular, respiratory and metabolic systems. The *Arg16Gly* (rs1042713) and *Gln27Glu* (rs1042714) polymorphisms have attracted the most interest.

In sport, the *Gly16* and *Glu27* alleles are nominally more frequent in speed–strength athletes than in sedentary individuals, which indicates their association with athlete status in these sports but does not allow conclusions about the effect of these alleles on athletic success. In endurance (aerobic cyclic) sports, the *Arg16Gly* polymorphism was associated with elite athlete status in one study, with the *Arg16* allele being more frequent in athletes; the *Arg16* allele has also been nominally associated with faster race times in runners. However, the meta-analysis [90] did not confirm an association of the *Arg16Gly* and *Gln27Glu* polymorphisms with the status of long-distance runners and road cyclists (the estimate was based on only two samples), and in study [20] only the *Gln27Glu* polymorphism was associated with athlete status (the *Glu27Glu* genotype was less frequent in athletes). In rhythmic gymnastics, the *Gly16Gly* genotype, which is associated with a lower BMI, is more frequent in high-level gymnasts than in controls. In judo, the association of the heterozygous *Arg16Gly* genotype with competitive success did not reach statistical significance.

The association of *ADRB2* with obesity remains controversial: it has been reported in some populations but not confirmed in others, including Japanese, Korean and Malaysian samples. The rs1042713 polymorphism has been associated with central fat accumulation, blood pressure, serum leptin and triglyceride levels and with insulin resistance rather than with overall obesity. The *Gln27Gln* homozygotes had a lower percentage of body fat than *Glu27Glu* homozygotes, and the *Glu27* allele has been associated with obesity and increased BMI in several studies, although a lower BMI in *Glu27* carriers has also been reported. In a meta-analysis, the *Glu27* allele was associated with obesity in Asians, Pacific Islanders and American Indians, but not in Europeans [46]. In individual studies, *ADRB2* polymorphisms were associated with differences in the response to weight-loss diets; a statistical interaction between *ADRB2* polymorphisms and the *LEPR* gene in relation to obesity risk in men has also been described.

An association of *ADRB2* with longevity has been described only in individual studies: the A-C haplotype of rs1042718 and rs1042719 was more frequent in long-lived individuals than in young people only in Han Chinese men, and this association requires confirmation in other populations. At advanced ages, interactions between *ADRB2* genotypes and regular exercise or social–leisure activities have been associated with better cognitive function and self-reported health, and carrying the minor alleles of rs1042718 or rs1042719 has been associated with fewer negative emotions. An increased risk of myocardial infarction in carriers of the *Glu27Glu* genotype (rs1042714) was found only in a subgroup with a specific compound genotype of the *ACE* gene, and data on the association of *ADRB2* with myocardial infarction are conflicting; therefore, the suggestion of an indirect link between *ADRB2* and life expectancy via cardiovascular disease remains a hypothesis.

In the respiratory system, the *Arg16Gly* and *Gln27Glu* polymorphisms have been associated in individual studies with the risk of asthma, uncontrolled asthma (*Arg16* allele and *Arg16Arg* genotype), the response to β2-agonist therapy and the risk of COPD exacerbations, although the direction of these associations differs between populations, and in a number of studies no associations were found. The *Glu27* variant has been associated with COPD exacerbations in individual European cohorts and with a reduced risk of COPD in an Egyptian sample; however, meta-analyses have not confirmed its association with COPD risk in Asian, Caucasian or African populations. The suggestion that a possible association of *ADRB2* with endurance is mediated by the cardiovascular and respiratory systems remains a hypothesis: in healthy non-athletes, the *Gly16Gly* genotype is associated with a greater stroke volume and cardiac output at rest and during short-term exercise, whereas differences in airway function have been found only after intense exercise, and the authors of the review [15] considered the effect of the genotype on the respiratory response to exercise to be insufficiently pronounced; in the studies reviewed, such data for athletes during prolonged exercise are lacking.

Overall, the results of the studies reviewed are inconsistent, which may be explained by differences in the populations studied, study design, ethnicity, measurement scales, nutrition and sample size. Almost none of the findings has been replicated in an independent sample, and about half of the studies included fewer than 500 participants (Table 1). Many associations were obtained without correction for multiple comparisons when several alleles, genotypes, haplotypes and subgroups were tested, and are therefore nominal. Moreover, all association studies reviewed are observational and do not demonstrate a causal effect of *ADRB2* variants on athletic or clinical phenotypes; this would require replication of the findings in large independent samples, as well as functional and interventional studies. Given the current state of knowledge, neither the *ADRB2* gene nor individual variants of other candidate genes can serve as a tool for predicting athletic performance or for selection, particularly in children [91]. The *ADRB2* gene should be regarded as a small-effect marker within multimarker genetic panels, which may in the future complement anthropometric, physiological and pedagogical criteria for sports orientation; the predictive value of such panels must be confirmed in large prospective studies.

## Figures and Tables

**Table 1:** Methodological quality of the *ADRB2* studies included in the review.

Study	Participants	Sample ≥ 500	Homogeneity	HWE	Phenotype	Replication	Quality
Section 3. Sport and physical performance							
Sawczuk et al. [7]	223 athletes/354 controls	+	+	no data	+	−	moderate
Tsianos et al. [8]	438 marathon runners	−	no data	+	+	−	low
Wolfarth et al. [9]	313 athletes/297 controls	+	+	+	+	−	moderate
Tringali et al. [13]	42 gymnasts/42 controls	−	no data	no data	+	−	low
Kwasniak et al. [19]	108 judoka, no controls	−	−	!	−	−	low
Varillas-Delgado et al. [20]	123 athletes/122 controls	−	+	+	+	−	moderate
Chou et al. [14]	559 adolescents	+	no data	no data	+	−	low
Tang et al. [16]	519 adults	+	+	no data	+	−	moderate
Section 4, Section 5 and Section 6 (number of studies meeting the criterion)							
4. Obesity and diet	17 studies, *n* = 75–4193	4	11	3	16	1	0/4/13
5. Longevity and cardiovascular disease	11 studies, *n* = 207–25,224	9	8	7	8	1	1/7/3
6. Asthma and COPD	12 studies, *n* = 36–8971	6	5	2	8	0	0/2/10

HWE, Hardy–Weinberg equilibrium; COPD, chronic obstructive pulmonary disease; +, criterion met; −, criterion not met; !, deviation from HWE; no data, considered as criterion not met. Quality: high, all 5 criteria met; moderate, 3–4 criteria met; low, 0–2 criteria met; for Section 4, Section 5 and Section 6, the numbers of studies of high/moderate/low quality are given. The meta-analysis [65] was not assessed; HWE for [64,66,67] is given according to its data.

**Table 2:** Studies of the associations of *ADRB2* polymorphisms with athlete status and performance, and meta-analyses.

Study	Sample	Variant	Phenotype	Main Result
Sawczuk et al. [7]	223 Polish athletes (123 endurance, 100 speed–strength); 354 contr.	*Arg16Gly*, *Gln27Glu*	Athlete status	*Gly16* and *Glu27* more frequent in speed–strength athletes (both *p* = 0.017, nominal); no association in the endurance group
Tsianos et al. [8]	438 mountain marathon participants	*Arg16Gly*	Race time	*Arg16*: faster race time only in a subgroup of 316 men (*p* = 0.01, nominal); no association in the total sample
Wolfarth et al. [9]	313 elite athletes from cyclic sports (North America, Finland, Germany); 297 contr.	*Arg16Gly*	Athlete status (VO_2_max ≥ 75 and ≤50 mL·kg^−1^·min^−1^)	Difference in genotype distribution (*p* = 0.03); *Gly16* carriers more frequent among controls (*p* = 0.009)
Tringali et al. [13]	42 gymnasts; 42 contr.	*Arg16Gly*	Gymnast status; BMI	*Gly16Gly* 42.8% versus 23.8%; *Arg16Arg* 7.1% versus 21.4%; *Gly16Gly*: lower BMI
Kwasniak et al. [19]	108 judoka (Poland, Croatia), no contr.	*Arg16Gly*	Competitive success	*Arg16Gly* more frequent in the strongest athletes; difference not significant (*p* = 0.06)
Varillas-Delgado et al. [20]	123 elite runners and cyclists; 122 contr. (Spain)	*Arg16Gly*, *Gln27Glu*	Athlete status	*Arg16Gly*: no differences (*p* = 0.943); *Glu27Glu* 13.8% versus 32.0% (*p* = 0.003); total score for 8 polymorphisms did not differ
Konopka et al. [90], meta-analysis	2 Spanish samples: 223 athletes; 222 contr.	*Arg16Gly*, *Gln27Glu*	Status of runners and road cyclists	OR 0.99 (0.67–1.46) for *Gly16Gly* and 1.29 (0.86–1.96) for *Gln27Gln*: association not confirmed
Psatha et al. [21], meta-analysis	107 articles, 37 genes	*Arg16Gly*, *Gln27Glu*	Athlete status	2 articles per polymorphism; no pooled analysis performed for *ADRB2*

BMI, body mass index; Contr., controls; OR, odds ratio; VO_2_max, maximal oxygen uptake; nominal, *p* < 0.05 without correction for multiple comparisons (Section 2.4). Study quality is presented in Table 1.

## Data Availability

No new data were created or analysed in this study. Data sharing is not applicable to this article.

## Supplementary Materials

The following supporting information can be downloaded at: https://www.mdpi.com/article/10.3390/genes17101311/s1, Table S1: Publication selection log, including the screening decision for each of the 1814 records retrieved from PubMed, the 5 studies identified by other methods and the classification of the 93 references.

## Abbreviations

The following abbreviations are used in this manuscript:

*ADRB2*	beta-2 adrenergic receptor gene
*Arg16Gly*	*ADRB2* polymorphism rs1042713 (c.46G>A, p.*Gly16Arg*)
*Gln27Glu*	*ADRB2* polymorphism rs1042714 (c.79G>C, p.*Glu27Gln*)
HWE	Hardy–Weinberg equilibrium
VO_2_max	maximal oxygen uptake
